# Shining a Light on America’s HIV Epidemic among Men who Have Sex with Men

**DOI:** 10.2196/publichealth.5864

**Published:** 2016-05-17

**Authors:** Ronald O Valdiserri

**Affiliations:** ^1^ Department of Health, Behavior and Society Bloomberg School of Public Health Johns Hopkins University Baltimore, MD United States

**Keywords:** HIV surveillance, HIV diagnosis, HIV prevalence, HIV incidence, men who have sex with men, gay and bisexual men, demography

It’s accepted as axiomatic that surveillance is the “cornerstone” of public health action [1]. Truthfully, without an accurate and timely assessment of population-specific health indicators and disease outcomes, public health policy makers and practitioners are often obliged to operate blindly at the margins of bias, misconception and ignorance. Historically, an accurate accounting of cases of the acquired immune deficiency syndrome (AIDS) – and later, human immunodeficiency virus (HIV) infections– was deemed so critical to America’s public health response to AIDS that surveillance was memorably referred to as the “conscience of the epidemic” [2]. Extending that metaphor to recent work by Rosenberg and colleagues [3], published in this issue of *JMIR Public Health and Surveillance*, reveals a truly troubled conscience, indeed.

Building off of work by Grey and colleagues [4], also recently published in *JMIR Public Health and Surveillance*, who estimated the size of MSM (men-who-have-sex-with-men) populations within states, counties and US metropolitan areas, Rosenberg et al. were able to generate estimates of the prevalence of HIV diagnosis and infection and the rate of new diagnoses at national, state, MSA and county levels. Their analysis revealed that “HIV infection is hyperendemic among MSM in many areas of the United States, particularly in the South” [3]. Relying on the UNAIDS definition of hyperendemicity, this means that in many MSM communities in the United States the prevalence of HIV infection is sustained at levels of 15% or even higher [5].

Although these researchers are not the first to call out the enormous burden of HIV disease among gay and bisexual men in the United States–especially among MSM who are black [6] – nor to highlight geographic HIV disparities in southern states [7], their results superimpose a mantle of urgency over an already critical public health problem. These findings beg the question “Why are we seeing such horrific HIV disparities among MSM in the US, especially among black MSM?” Hyperbole aside, epidemiologists note “if black MSM in the United States formed a country…it would have the highest HIV prevalence on the globe” [8]. And, more to the point, what can we do to remediate the circumstances fueling these appalling outcomes?

The answer, of course, is *action*. Public health surveillance without responsive public health action is, at best, wasted effort and at worst, negligence. The action must be timely and commensurate with the scale and scope of the problem but, above all else, it must be informed. This last statement brings us to another well-accepted (if hard won) axiom gained from our decades of interaction with this virus: there is no “magic bullet” that will end the AIDS epidemic.  In other words, there is no *single* action or intervention that, once undertaken, will wondrously wipe away these disparities. We have to accept the inescapable reality that successfully confronting HIV among gay and bisexual men in the United States, including attending to the special needs of MSM of color, will require us to mount a comprehensive array of interventions, including structural interventions and policy changes which address the social determinants underpinning the aforementioned health disparities.  In the words of Teutsch and Fielding in their essay “Rediscovering the Core of Public Health,” while the principles of public health “remain the same” the “solutions are different”; namely, to be successful, public health cannot rely solely on biomedical approaches but must also “create healthier communities” [9].

What specific actions must we undertake, as public health leaders, to reduce HIV disparities and create healthier communities for same gender-loving men?  A sensible starting place is America’s National HIV/AIDS Strategy, updated and released in 2015 [10]. Under Goal 3, “Reducing HIV-Related Disparities and Health Inequities,” we find the following recommended actions: expand services, support engagement in care, scale-up programs that address social determinants of health (SDOH), and mobilize communities to reduce stigma. Each one of these recommendations represents a key component of the comprehensive response necessary to quell the HIV epidemic among MSM in the United States.

Acknowledging the reality that many MSM living in the United States face barriers to accessing HIV prevention and clinical services [11,12] should not be misconstrued to imply that we are powerless to confront the disparities described by Rosenberg and his colleagues. In truth, compared to the earliest days of the HIV epidemic, we possess a wealth of knowledge about the virus and a robust armamentarium of clinical and public health interventions to treat and prevent HIV infection. But, in the oft-quoted words of Goethe, “Knowing is not enough, we must apply; willing is not enough, we must do.”

No intervention, regardless of efficacy, will demonstrate effectiveness if it’s not implemented properly or to scale.  In its most recent assessment of HIV prevention services provided by state and local health departments, the National Alliance of State and Territorial AIDS Directors (NASTAD) found that “a lack of adequate HIV prevention funding across all program areas examined presents a major challenge for health department programs” [13].  Consistent with NASTAD’s findings is a model published by Holtgrave and colleagues estimating that unmet HIV service needs among black MSM in the United States approached nearly $2.5 billion in 2011 US dollars [14]. Clearly, as in any venture—health or otherwise—strategic investments pay dividends, in this case, reductions in preventable disease and death. Simply stated, if we want to end the HIV epidemic among MSM in the United States, we must increase our public health investment in needed prevention, care and social services. 

Essential though it may be, investing additional resources into expanding HIV services for MSM is not the only necessary action we must undertake to resolve these disparities. Consider the findings of Oldenburg and her colleagues who documented a strong statistical association between state level structural stigma directed toward LGBT (lesbian, gay, bisexual and transgender) populations and “increased sexual risk behavior, decreased awareness and use of antiretroviral chemoprophylaxis and decreased comfort discussing sexual behavior with primary care providers” [11]. This is but one example of a burgeoning literature shining a harsh light on the insidious connection between stigma and suboptimal health outcomes.  Indeed, if we seek to reduce HIV-related disparities among MSM populations (and other vulnerable groups) it is imperative that we support models of prevention and care that knowingly address the diversity of factors that interact to shape what we call “health” [15]. This brings us back, full-circle, to our shared mission as public health leaders: “fulfilling society’s interest in assuring conditions in which people can be healthy” [16]. Assuring conditions in which MSM (whether gay identified or not) can be healthy–in this case free of HIV infection or continuously virally suppressed–requires that we move beyond strictly biomedical approaches. Even when those biomedical approaches are impressively effective, as in the case of PrEP (pre-exposure prophylaxis), provider attitudes can have a dampening effect on uptake, the daily realities of unemployment and unstable housing can minimize their relative importance among potential consumers and policies related to health care financing and reimbursement can inadvertently block access for those populations most likely to benefit from these amazing biomedical advances. 

Analyses that continue to document the tremendous burden of HIV among MSM and call-out sub-populations and geographic areas that are particularly hard-hit may be disturbing to acknowledge. But every one of us–epidemiologist or not– understands that a troubled conscience will only grow worse if we continue to ignore the problem at hand rather than pursue those actions required to resolve the situation.
